# Delineating blood DNA methylation biomarkers for Alzheimer's disease

**DOI:** 10.1002/alz.70646

**Published:** 2025-09-02

**Authors:** Ehsan Pishva, Lars Bertram, Katie Lunnon

**Affiliations:** ^1^ Department of Clinical and Biomedical Sciences Faculty of Health and Life Sciences University of Exeter Exeter UK; ^2^ Department of Psychiatry and Neuropsychology, School for Mental Health and Neuroscience (MHeNs), Faculty of Health, Medicine and Life Sciences (FHML) Maastricht University Maastricht the Netherlands; ^3^ Lübeck Interdisciplinary Platform for Genome Analytics (LIGA) University of Lübeck Lübeck Germany

**Keywords:** Alzheimer's disease, biomarker, blood, cerebrospinal fluid, DNA methylation, epigenetics, epigenome‐wide association study, methylation quantitative trait loci, neurofilament light chain, protein quantitative trait loci, YKL‐40

## Abstract

Epigenetic mechanisms act as mediators of genetic and environmental influences. In Alzheimer's disease, blood‐based DNA methylation signatures are increasingly being explored as minimally invasive peripheral biomarkers. We previously reported associations between blood DNA methylation in the *CHI3L1* gene (encoding YKL‐40) and cerebrospinal fluid (CSF) levels of YKL‐40, a marker of neuroinflammation. These findings have now been replicated in an independent study (Kaleck et al. 2025), reinforcing their robustness and biological relevance. We also reported associations between DNA methylation and CSF neurofilament light chain levels, a marker of axonal damage, which were not independently replicated in the analyses by Kaleck et al. Several factors may have contributed to this inconsistency in results, which we discuss in detail as they are relevant to future DNA methylation‐based studies in the field. Together, these findings highlight both the potential and the complexity of identifying consistent blood‐based epigenomic signatures for neurodegenerative processes. They also underscore the importance of harmonizing methodologies across studies to improve reproducibility and, eventually, to yield meaningful biomarkers allowing an early detection of this devastating disease.

## 1 INTRODUCTION

Epigenetic mechanisms act as mediators of genetic and environmental influences and robust DNA methylation (DNAm) patterns have been identified in Alzheimer's disease (AD) brain through epigenome‐wide association studies (EWAS).^1^, ^2^, ^3^, ^4^, ^5^, ^6^, ^7^, ^8^, ^9^, ^10^, ^11^, ^12^, ^13^, ^14^ More recently, blood‐based DNAm signatures are being investigated as minimally invasive biomarkers for AD,^15^, ^16^, ^17^, ^18^ including those associated with cerebrospinal fluid (CSF) biomarkers of neurodegeneration.^19^, ^20^

In a recent study using the EMIF‐AD cohort (*N* = 886) we examined associations between blood DNAm and 15 CSF biomarkers of AD and neurodegeneration.^19^ Our most robust finding was elicited by five Bonferroni‐significant differentially methylated positions (DMPs) in the *CHI3L1* gene (encoding YKL‐40) associated with CSF YKL‐40, a biomarker of glial activation and neuroinflammation.^21^ These associations were independently replicated by Kaleck et al.,^22^ who also highlighted two additional DMPs in *CHI3L1* that we had reported at nominal significance.

Together, these findings reinforce the robustness of the *CHI3L1* DNAm–protein association across independent cohorts and analytical pipelines. Notably, our study also showed that *CHI3L1* DNAm is influenced by a *cis*‐methylation quantitative trait locus (mQTL) mapping to a single nucleotide polymorphism (SNP) previously identified in a genome‐wide association study (GWAS) of CSF YKL‐40 levels.^23^ We also demonstrated a correlation of blood *CHI3L1* DNAm and plasma YKL‐40 levels, suggesting that DNAm may mediate genetic regulation of YKL‐40 in CSF and blood. These findings highlight the potential of targeting *CH13L1* DNAm to regulate brain YKL‐40 levels, which could modulate neuroinflammation.

We also reported seven Bonferroni‐significant DMPs associated with CSF neurofilament light chain (NfL),^19^ a biomarker of neuro‐axonal damage.^24^ However, Kaleck et al. did not replicate these findings in their meta‐analysis. Several factors may account for this discrepancy, highlighting important considerations for future studies.

## 2 STUDY DESIGN FACTORS

Our NfL EWAS used data from 662 individuals in EMIF‐AD, a harmonized pan‐European cohort. In contrast, the Kaleck et al. study included 448 and 276 individuals from the DELCODE (Germany) and GR@ACE (Spain) cohorts, respectively. A key difference, therefore, is that our data came from a single large cohort, while Kaleck et al. meta‐analyzed two smaller cohorts. Although meta‐analyses using two datasets are possible, there are limitations as it may yield unstable and in parts unreliable estimates of between‐study heterogeneity.^25^ This undermines a core objective of meta‐analysis, which is quantifying and understanding variability across datasets. In such cases, observed differences may merely reflect random error rather than true heterogeneity, making interpretation problematic. Furthermore, if one study is substantially larger, it can disproportionately influence the pooled effect size, effectively reducing the meta‐analysis to a weighted summary of that single dominant study.^26^ This may explain why one of our NfL differentially methylated regions (DMRs) was replicated in GR@ACE (chr5:23507349‐23507644) but not in DELCODE and, accordingly, not in their meta‐analysis.

## 3 TECHNICAL FACTORS

EMIF‐AD used a commercial enzyme‐linked immunosorbent assay (ELISA; NF‐light ELISA, UmanDiagnostics^27^) to quantify NfL, whereas the DELCODE and GR@ACE cohorts used the Proximity Extension Assay from Olink. These platforms differ in antibody sensitivity and specificity. Moreover, to minimize batch effects, all EMIF‐AD samples were measured in one round of experiments performed in one laboratory. Unfortunately, Kaleck et al. provided no information on the experimental layout for NfL measurements.

For DNAm data processing we used the watermelon package,^28^ implementing stringent quality control (QC), excluding samples and probes based on detection *P* values and bead counts. After quantile normalization, three samples were excluded as principal component (PC) analysis outliers, though we retained outliers at individual CpG sites, as these may reflect potential mQTLs relevant to disease biology—an approach supported by our YKL‐40 analysis. While four of the seven Bonferroni‐significant NfL DMPs did contain some outliers, and the significance was attenuated when these were removed, the remaining three Bonferroni‐significant loci did not. The stringent QC pipeline in the Smith et al. study resulted in 882 high‐quality samples (662 with NfL measurements) and 861,666 CpG probes retained for analysis. In contrast, Kaleck et al. reported using the meffil package but did not specify sample or probe‐level inclusion/exclusion criteria, nor normalization method. Furthermore, in the absence of access to their analytical code in their manuscript, it is not possible to assess how their computational pipeline fully compares to ours (available at https://github.com/UoE‐Dementia‐Genomics/EMIF_Biomarkers_Methylation).

Both studies used multiple linear regression models, adjusting for age, sex, and estimated cell composition. However, additional covariates differed: we included bisulfite plate, study center, and four genetic PCs, while Kaleck et al. adjusted for smoking (estimated via cg05575921 methylation) and an unspecified number of surrogate variables.

## 4 BIOLOGICAL FACTORS

It is also possible that other sample‐specific confounding factors contributed to the lack of replication. As outlined above, there are population‐level differences between the studies: our study was pan‐European, whereas Kaleck et al. drew on regional cohorts from Germany and Spain. Genetic variability across European populations is well documented and, if not accounted for, can influence association results.^29^ In our study, we attempted to mitigate this by adjusting for both study center and genetic PCs. While the Kaleck et al. meta‐analysis included two distinct populations, it did not report on (adjustment for) genetic ancestry, making it difficult to assess the extent to which population substructure may have influenced their findings. Last, there may also be genuine biological differences between cohorts, such as subtle variations in AD or mild cognitive impairment stage, which is relevant for an early AD marker such as NfL.^30^

## 5 CONCLUSION

The most compelling and consistent finding across both studies is the *CHI3L1*‐YKL‐40 association, reinforcing the robustness of this signal. While some variation is genetically driven, *CHI3L1* DNAm still shows meaningful inter‐individual differences and correlates with protein levels, suggesting potential clinical relevance.

The non‐replication of the NfL association may stem from a number of differences in the studies. We also cannot exclude the possibility of a false‐positive finding in our study. Nonetheless, this underscores the need for larger, harmonized, and well‐powered EWASs to yield robust and meaningful associations.

Together, these studies highlight both the promise and complexity of identifying consistent blood‐based methylomic signatures of neurodegeneration. They provide an important foundation for future collaborative work toward developing clinically useful epigenomic AD biomarkers.

## CONFLICT OF INTEREST STATEMENT

All authors declare that they have nothing to disclose. Author disclosures are available in the supporting information

## Supporting information



Supporting Information

## References

[1] De Jager PL , Srivastava G , Lunnon K , et al. Alzheimer's disease: early alterations in brain DNA methylation at ANK1, BIN1, RHBDF2 and other loci. Nat Neurosci. 2014;17:1156‐1163. doi:10.1038/nn.3786 25129075 PMC4292795

[2] Lunnon K , Smith R , Hannon E , et al. Methylomic profiling implicates cortical deregulation of ANK1 in Alzheimer's disease. Nat Neurosci. 2014;17:1164‐1170. doi:10.1038/nn.3782 25129077 PMC4410018

[3] Lardenoije R , Roubroeks JAY , Pishva E , et al. Alzheimer's disease‐associated (hydroxy)methylomic changes in the brain and blood. Clinical epigenetics. 2019;11:164. doi:10.1186/s13148-019-0755-5 31775875 PMC6880587

[4] Smith AR , Smith RG , Pishva E , et al. Parallel profiling of DNA methylation and hydroxymethylation highlights neuropathology‐associated epigenetic variation in Alzheimer's disease. Clinical epigenetics. 2019;11:52. doi:10.1186/s13148-019-0636-y 30898171 PMC6429761

[5] Smith RG , Hannon E , De Jager PL , et al. Elevated DNA methylation across a 48‐kb region spanning the HOXA gene cluster is associated with Alzheimer's disease neuropathology. Alzheimers Dement. 2018;14:1580‐1588. doi:10.1016/j.jalz.2018.01.017 29550519 PMC6438205

[6] Pishva E , Creese B , Smith AR , et al. Psychosis‐associated DNA methylomic variation in Alzheimer's disease cortex. Neurobiol Aging. 2020;89:83‐88. doi:10.1016/j.neurobiolaging.2020.01.001 32007278

[7] Kouhsar M , Weymouth L , Smith AR , et al. A brain DNA co‐methylation network analysis of psychosis in Alzheimer's disease. Alzheimers Dement. 2025;21:e14501. doi:10.1002/alz.14501 39936280 PMC11815327

[8] Sommerer Y , Dobricic V , Schilling M , et al. Entorhinal cortex epigenome‐wide association study highlights four novel loci showing differential methylation in Alzheimer's disease. Alzheimers Res Ther. 2023;15:92. doi:10.1186/s13195-023-01232-7 37149695 PMC10163801

[9] Li QS , Sun Y , Wang T . Epigenome‐wide association study of Alzheimer's disease replicates 22 differentially methylated positions and 30 differentially methylated regions. Clinical epigenetics. 2020;12:149. doi:10.1186/s13148-020-00944-z 33069246 PMC7568396

[10] Gasparoni G , Bultmann S , Lutsik P , et al. DNA methylation analysis on purified neurons and glia dissects age and Alzheimer's disease‐specific changes in the human cortex. Epigenetics Chromatin. 2018;11:41. doi:10.1186/s13072-018-0211-3 30045751 PMC6058387

[11] Watson CT , Roussos P , Garg P , et al. Genome‐wide DNA methylation profiling in the superior temporal gyrus reveals epigenetic signatures associated with Alzheimer's disease. Genome medicine. 2016;8:5. doi:10.1186/s13073-015-0258-8 26803900 PMC4719699

[12] Smith RG , Pishva E , Shireby G , et al. A meta‐analysis of epigenome‐wide association studies in Alzheimer's disease highlights novel differentially methylated loci across cortex. Nat Commun. 2021;12:3517. doi:10.1038/s41467-021-23243-4 34112773 PMC8192929

[13] Shireby G , Dempster EL , Policicchio S , et al. DNA methylation signatures of Alzheimer's disease neuropathology in the cortex are primarily driven by variation in non‐neuronal cell‐types. Nat Commun. 2022;13:5620. doi:10.1038/s41467-022-33394-7 36153390 PMC9509387

[14] Zhang L , Silva TC , Young JI , et al. Epigenome‐wide meta‐analysis of DNA methylation differences in prefrontal cortex implicates the immune processes in Alzheimer's disease. Nat Commun. 2020;11:6114. doi:10.1038/s41467-020-19791-w 33257653 PMC7704686

[15] Roubroeks JAY , Smith AR , Smith RG , et al. An epigenome‐wide association study of Alzheimer's disease blood highlights robust DNA hypermethylation in the HOXB6 gene. Neurobiol Aging. 2020;95:26‐45. doi:10.1016/j.neurobiolaging.2020.06.023 32745807 PMC7649340

[16] Li QS , Vasanthakumar A , Davis JW , et al. Association of peripheral blood DNA methylation level with Alzheimer's disease progression. Clinical epigenetics. 2021;13:191. doi:10.1186/s13148-021-01179-2 34654479 PMC8518178

[17] Vasanthakumar A , Davis JW , Idler K , et al. Harnessing peripheral DNA methylation differences in the Alzheimer's Disease Neuroimaging Initiative (ADNI) to reveal novel biomarkers of disease. Clinical epigenetics. 2020;12:84. doi:10.1186/s13148-020-00864-y 32539856 PMC7294637

[18] Zhang W , Young JI , Gomez L , et al. Blood DNA methylation signature for incident dementia: evidence from longitudinal cohorts. Alzheimer's & dementia : the journal of the Alzheimer's Association. 2025;21:e14496. doi:10.1002/alz.14496 PMC11936765 40133250

[19] Smith RG , Pishva E , Kouhsar M , et al. Blood DNA methylomic signatures associated with CSF biomarkers of Alzheimer's disease in the EMIF‐AD study. Alzheimers Dement. 2024;20:6722‐6739. doi:10.1002/alz.14098 39193893 PMC11485320

[20] Zhang W , Young JI , Gomez L , et al. Distinct CSF biomarker‐associated DNA methylation in Alzheimer's disease and cognitively normal subjects. Alzheimers Res Ther. 2023;15:78. doi:10.1186/s13195-023-01216-7 37038196 PMC10088180

[21] Olsson B , Lautner R , Andreasson U , et al. CSF and blood biomarkers for the diagnosis of Alzheimer's disease: a systematic review and meta‐analysis. Lancet Neurol. 2016;15:673‐684. doi:10.1016/S1474-4422(16)00070-3 27068280

[22] Kaleck T , et al. Replication of blood DNA methylomic signatures associated with cerebrospinal fluid levels of YKL‐40 and NfL biomarkers. Alzheimer's & dementia : the journal of the Alzheimer's Association. 2025. 10.1002/alz.70647 PMC12405059 40898416

[23] Hong S , Prokopenko D , Dobricic V , et al. Genome‐wide association study of Alzheimer's disease CSF biomarkers in the EMIF‐AD Multimodal Biomarker Discovery dataset. Translational psychiatry. 2020;10:403. doi:10.1038/s41398-020-01074-z 33223526 PMC7680793

[24] Khalil M , Teunissen CE , Lehmann S , et al. Neurofilaments as biomarkers in neurological disorders—towards clinical application. Nat Rev Neurol. 2024;20:269‐287. doi:10.1038/s41582-024-00955-x 38609644

[25] Borenstein M , Hedges LV , Higgins JPT , Rothstein HR . Introduction to Meta‐Analysis. John Wiley & Sons, Ltd; 2009.

[26] Higgins JP , Whitehead A , Simmonds M . Sequential methods for random‐effects meta‐analysis. Stat Med. 2011;30:903‐921. doi:10.1002/sim.4088 21472757 PMC3107948

[27] Zetterberg H , Skillbäck T , Mattsson N , et al. Association of Cerebrospinal Fluid Neurofilament Light Concentration With Alzheimer Disease Progression. JAMA Neurol. 2016;73:60‐67. doi:10.1001/jamaneurol.2015.3037 26524180 PMC5624219

[28] Pidsley R , Y Wong CC , Volta M , Lunnon K , Mill J , Schalkwyk LC . A data‐driven approach to preprocessing Illumina 450K methylation array data. Bmc Genomics [Electronic Resource]. 2013;14:293. doi:10.1186/1471-2164-14-293 23631413 PMC3769145

[29] Novembre J , Johnson T , Bryc K , et al. Genes mirror geography within Europe. Nature. 2008;456:98‐101. doi:10.1038/nature07331 18758442 PMC2735096

[30] Hofmann A , Häsler LM , Lambert M , et al. Comparative neurofilament light chain trajectories in CSF and plasma in autosomal dominant Alzheimer's disease. Nat Commun. 2024;15:9982. doi:10.1038/s41467-024-52937-8 39557867 PMC11574007

